# Interleukin-6 and Hypoxia Synergistically Promote EMT-Mediated Invasion in Epithelial Ovarian Cancer via the IL-6/STAT3/HIF-1*α* Feedback Loop

**DOI:** 10.1155/2023/8334881

**Published:** 2023-02-13

**Authors:** Tongshuo Zhang, Jing Yang, Yang Sun, Jiangnan Song, Dandan Gao, Suhui Huang, Aibo Pang, Jianhui Zhang, Junhong Wang, Yue Wang, Yanqiu Li

**Affiliations:** ^1^School of Integrative Medicine, Tianjin University of Traditional Chinese Medicine, Tianjin 301617, China; ^2^Department of Pathogenic Biology, Logistics University of Chinese People's Armed Police Force (PAP), Tianjin 300309, China; ^3^Department of Clinical Laboratory and Pathology, Jiangsu Provincial Corps Hospital of PAP, Yangzhou 225003, China; ^4^Department of Gynaecology and Obstetrics, Characteristic Medical Center of PAP, Tianjin 300162, China; ^5^Medical School of Chinese People's Liberation Army (PLA), Chinese PLA General Hospital, Beijing 100853, China; ^6^Department of Disease Control and Prevention, Tibetan Armed Police Force Hospital, Lhasa, Tibet 850000, China; ^7^Affiliated Beichen Hospital, Tianjin University of Traditional Chinese Medicine, Tianjin 300400, China; ^8^Department of Clinical Laboratory, Characteristic Medical Center of PAP (Formerly Affiliated Hospital of Logistics University of PAP), Tianjin 300162, China

## Abstract

Extensive peritoneal spread and capacity for distant metastasis account for the majority of mortality from epithelial ovarian cancer (EOC). Accumulating evidence shows that interleukin-6 (IL-6) promotes tumor invasion and migration in EOC, although the molecular mechanisms remain to be fully elucidated. Meanwhile, the hypoxic microenvironment has been recognized to cause metastasis by triggering epithelial–mesenchymal transition (EMT) in several types of cancers. Here, we studied the synergy between IL-6 and hypoxia in inducing EMT in two EOC cell lines, A2780 cells and SKOV3 cells. Exogenous recombination of IL-6 and autocrine production of IL-6 regulated by plasmids both induced EMT phenotype in EOC cells characterized by downregulated E-cadherin as well as upregulated expression of vimentin and EMT-related transcription factors. The combined effects of IL-6 and hypoxia were more significant than those of either one treatment on EMT. Suppression of hypoxia-inducible factor-1*α* (HIF-1*α*) before IL-6 treatment inhibited the EMT phenotype and invasion ability of EOC cells, indicating that HIF-1*α* occupies a key position in the regulatory pathway of EMT associated with IL-6. EMT score was found positively correlated with mRNA levels of IL-6, signal transducer and activator of transcription 3 (STAT3), and HIF-1*α*, respectively, in 489 ovarian samples from The Cancer Genome Atlas dataset. Next, blockade of the abovementioned molecules by chemical inhibitors reversed the alteration in the protein levels of EMT markers induced by either exogenous or endogenous IL-6. These findings indicate a positive feedback loop between IL-6 and HIF-1*α*, and induce and maintain EMT phenotype through STAT3 signaling, which might provide a novel rationale for prognostic prediction and therapeutic targets in EOC.

## 1. Introduction

Metastasis and infiltration often occur early in epithelial ovarian cancer (EOC) and determine the progress and prognosis of EOC patients [1]. Epithelial–mesenchymal transition (EMT), which is a major feature of metastatic cells, is a conserved cellular program that alters cell shape, adhesion, and movement [2]. It is well established that the tumor microenvironment can induce EMT through paracrine signals involving various EMT inducers, such as transforming growth factor-beta [3]. Identification of EMT inducers will contribute to discovering new molecular targets to prevent and control metastasis of EOC in the clinic [4, 5]. Epithelial cells undergoing EMT typically exhibit decreased expression of the epithelial cell marker E-cadherin and increased expression of the mesenchymal marker vimentin and core transcription factors, which promote the expression of EMT markers, such as Twist1 and Snail [6].

Chronic inflammation is demonstrably capable of fostering neoplastic progression and, as such, is regarded as the seventh hallmark of cancer [7]. Interleukin-6 (IL-6) is an inflammatory cytokine with multiple functions [8]. Previous studies showed that increased IL-6 in the serum and ascites fluid in EOC patients are associated with advanced disease stage and poor clinical outcome [9–11]. Our prior research indicated that IL-6 confers on EOC cell traits including proliferation, adhesion, and invasion [12], although the mechanism underlying the aggressive behavior produced by IL-6 remains to be fully elucidated [13]. In spite of the wide range of evidence for EMT induced by IL-6 signaling in certain tumor types [14–16], the links between IL-6 and EMT in EOC are poorly documented at present. Sullivan et al. [17] identified IL-6 as potent inducer of EMT responsible for metastasis in breast cancer. Gyamfi et al. [18] also demonstrated that IL-6 secreted by adipocytes induced EMT in breast cancer cells. In light of the many similarities between ovarian and breast cancer, such as hormone response and estrogen receptor distribution [19, 20], IL-6 appears to play the same role in regulating EMT in EOC as that in breast cancer.

Representing another cancer hallmark, hypoxia exists in solid tumors and gives rise to ulterior malignity and metastasis by modifying cellular metabolism and triggering varied molecular signaling pathways. Numerous evidences have revealed the widespread presence of hypoxia-inducible factor-1*α* (HIF-1*α*), the core transcription factor that regulates oxygen homeostasis, in the tumor microenvironment, and pointed to its significance for the regulation of cellular receptors associated with inflammation response [21–23]. Experiments in a series of cancers demonstrated that HIF-1*α* provokes EMT phenotype or characteristics by directly augmenting EMT-related transcription factors like zinc finger E-box binding homeobox (ZEB), Slug, Twist, and Snail [24, 25]. Additionally, inflammatory cytokines modulated by hypoxia, such as tumor necrosis factor-alpha and IL-1*β*, also involve potential EMT-associated signaling processes [26].

To our knowledge, no study has been reported to investigate the inducements of invasion or EMT in EOC based on combining IL-6 and hypoxia within the tumor microenvironment. Recently, we have confirmed the formation of IL-6/STAT3/HIF-1*α* autocrine signaling loop in ovarian cancer cells *in vitro* and *in vivo*, which confers chemoresistance against cisplatin to ovarian cancer [27]. IL-6/STAT3/HIF-1*α* loop fulfills critical roles in the cross-communication between Il-6 and hypoxia in EOC; therefore, we supposed the loop may enhance the responsiveness to IL-6 and hypoxia during the acquisition of EMT.

This study explored the synergy between IL-6 and hypoxia and its delicate mechanism in EMT-mediated invasion. Our data demonstrated the synergistic inducing EMT effect of IL-6 combined with hypoxia via the IL-6/STAT3/HIF-1*α* autocrine loop, which contributes to understanding cancer progression and invasiveness, and developing a feasible therapeutic strategies for EOC.

## 2. Materials and Methods

### 2.1. Cell Lines, Cell Culture, and Cell Transfection

Human ovarian cancer cell lines (A2780, SKOV3, CAOV-3, and ES-2) were purchased from the (American Type Culture Collection, Manassas, VA, USA). A2780 and ES-2 cells were grown in RPMI 1640 (Life Technologies, Inc., Gaithersburg, MD, USA) supplemented with 10% fetal bovine serum (FBS; Life Technologies, Inc.), SKOV-3 and CAOV-3 cells were cultured in Dulbecco's modified Eagle's medium (Life Technologies, Inc.) containing 15% FBS.

A2780 cells were stably transfected with a plasmid containing the sense IL-6 sequence (A2780/ssIL-6), and SKOV3 cells were stably transfected with a plasmid containing the antisense IL-6 sequence (SKOV3/asIL-6) as previously described in Wang et al. [28]. The A2780/ssIL-6 stable cell line overexpressing IL-6 and the SKOV-3/asIL-6 stable cell lines with repressed IL-6 production were cloned and validated [28]. The empty vector pcDNA3.1(+) was also transfected into A2780 (A2780/pcDNA3.1(+)) or SKOV3 (SKOV3/pcDNA3.1(+)) cells as negative controls. A2780 cells were also transiently transfected with a plasmid encoding HIF-1*α* (A2780/ssHIF-1*α*), and SKOV3 cells were transiently transfected with a plasmid silencing HIF-1*α* (SKOV3/HIF-1*α*-shRNA) with Lipofectamine™2000 (Invitrogen, Carlsbad, CA, USA) according to the manufacturer's protocol. The empty vector was transiently transfected into these two cell lines as negative controls (A2780/pCMVh-HA and SKOV3/scramble shRNA).

Cells were cultured at 37°C in a humidified atmosphere containing 5% CO_2_ and exposed to normoxic (21% O_2_), hypoxic (1% O_2_), or simulated hypoxic (CoCl_2_) conditions. Hypoxic conditions were maintained using a modular incubator chamber (Billups-Rothenberg, Inc., Del Mar, CA, USA) with 5% CO_2_ and 1% O_2_ balanced with N_2_ gas. CoCl_2_, as a chemical hypoxia mimic was obtained from Sigma (San Francisco, CA, USA) and dissolved in D-Hank's balanced salt solution (Solarbio Technology Co., Beijing, China). The optimal concentrations of CoCl_2_ for stimulated hypoxic conditions in A2780 cells (50 *μ*M) and SKOV3 cells (100 *μ*M) were defined in pilot experiments (data not shown). The Materials and Methods section should contain sufficient details so that all procedures can be repeated. It may be divided into headed subsections if several methods are described.

### 2.2. Cytokine and Inhibitor Treatments

Recombinant human IL-6 (Peprotech, Rocky Hill, NJ, USA) was used to evaluate the effect of exogenous IL-6 on the EMT phenotype of A2780 and SKOV3 cells based on a previously established dosing regimen (A2780 cells were cultured in the presence of IL-6 (50 ng/ml) for 24 hours, whereas SKOV3 cells were cultured in the presence of IL-6 (10 ng/ml) for 48 hours). Pharmacological inhibitors of STAT3 (AG490) and HIF-1*α* (YC-1) were purchased from Cayman Chemical (Ann Arbor, MI, USA). Both A2780 and SKOV-3 cells were treated with YC-1 (10 *μ*M) for 1 hour prior to the addition of exogenous IL-6. Cells were pretreated with AG490 (50 *μ*M) 30 minutes prior to the addition of IL-6.

### 2.3. Real-Time Quantitative Polymerase Chain Reaction

Total RNA was isolated from EOC cell lines and nude mouse tissue with TRIzol (Invitrogen, San Diego, CA, USA) according to the manufacturer's protocol. RNA was reverse transcribed to complementary DNA (cDNA) using the PrimeScript Reverse Transcription Kit (CWBIO, Beijing, China). The mRNA levels were measured by real-time quantitative polymerase chain reaction (RT-qPCR) using SYBR Green PCR Master Mix (TransGen Biotech, Beijing, China), which was performed on the BIO-RAD CFX96 touch RT-qPCR system (Bio-Rad Laboratories, Inc., Irvine City, CA, USA). The relative expression of the target genes was normalized to the expression of *β*-actin using the 2^−*ΔΔ*Ct^ method. All experiments were repeated at least three times. The primers were synthesized by Synbio Tech Co., Ltd. (Suzhou, China) and listed below: E-cadherin, 5′-CACCACGTACAAGGGTCAGG-3′ (forward) and 5′-TGCATCTTGCCAGGTCCTTTA-3′ (reverse); vimentin, 5′-GGACCAGCTAAC CAACGACA-3′ (forward) and 5′-AAGGTCAAGACGTGCCAGAG-3′ (reverse); Twist1, 5′-ATTCAAAGAAAACAGGGCGTGG-3′ (forward) and 5′-CCTTTCAGTGGCTGATTGGAC-3′ (reverse); Snail, 5′-GGAGTGGTTCTTCTTCTGCGCTA-3′ (forward) and 5′-GGGCTGCTGGAAGGTAAACT-3′ (reverse); and *β*-actin, 5′-GCACTCTTCCAGCCTTCCTT-3′ (forward) and 5′-AATG CCAGGGTACATGGTGG-3′ (reverse).

### 2.4. Western Blot Analysis

Total proteins were prepared by complete cell lysis (Keygen Biotech, Nanjing, Jiangsu, China) with protease and phosphatase inhibitors. Proteins were separated by sodium dodecyl sulfate–polyacrylamide gel electrophoresis and then electrotransferred onto polyvinylidene difluoride membranes. After blocking with 5% bovine serum albumin (BSA)-phosphate buffered solution (PBS) for 1 hour at room temperature, membranes were incubated overnight at 4°C with specific primary antibodies diluted in 0.2% Tween-PBS: rabbit anti-E-cadherin (1 : 500; Cell Signalling, Danvers, MA, USA), mouse anti-vimentin (1 : 500; Huabio, Hangzhou, China), rabbit anti-Twist1 (1 : 500; Signalway Antibody, College Park, MD, USA), rabbit anti-Snail (1 : 500; Cell Signalling), mouse anti-HIF-1*α* (1 : 1000; Abcam, Cambridge, UK), and mouse anti-*β*-actin (1 : 8000; Santa Cruz Biotechnology, Santa Cruz, CA, USA). Next, membranes were incubated for 1 hour at 37°C with corresponding secondary Peroxidase Horseradish (HRP)-conjugated antibodies (1 : 8000; Sigma, Santa Clara, CA, USA). The proteins were subsequently visualized using a chemiluminescence detection system (ECL Plus Western Blotting Detection System; Amersham Biosciences, Foster City, CA, USA). The intensity of the relative bands was assessed by densitometric analysis (ImageJ software program). *β*-Actin antibodies were used as controls for equal protein loading.

### 2.5. Immunofluorescence

After the pretreatment of exogenous IL-6, EOC cells were plated into chamber slides in six-well plates at a density of 10 × 10^5^ cells/well for A2780 and 6 × 10^5^ cells/well for SKOV3. The next day, cells were fixed onto slides within 4% ice-cold paraformaldehyde for 15 minutes and then washed three times (5 minutes/wash) in PBS. Slides were blocked with 5% BSA-PBS for 1 hour at room temperature. Subsequently, slides were incubated overnight at 4°C with specific primary antibodies diluted in 0.2% Tween-PBS: rabbit anti-E-cadherin (1 : 200; Cell Signalling) and mouse anti-vimentin (1 : 200; Huabio). Slides were incubated for 1 hour at 37°C with corresponding secondary Dylight594-conjugated antibody (1 : 1,000; Abcam) and Dylight488-conjugated antibody (1 : 1,000; Abcam). Nuclei were counterstained with the fluorescent dye 4,6-diamino-2-phenyl indole (DAPI) (0.25 mg/ml; Invitrogen) before the slides were mounted onto glasses with SlowFade Gold antifade reagent (Invitrogen). Immunofluorescences were observed, and images were captured under the Nikon Eclipse 90i fluorescence microscope (Nikon, Tokyo, Japan).

### 2.6. Invasion Assay

The invasive ability of cells was detected by an *in vitro* transwell chamber invasion assay. After transfection or treatment, EOC cells were seeded into the upper chamber of a polycarbonate membrane transwell chamber at a density of 4 × 10^5^ cells/well for A2780 and 3 × 10^5^ cells/well for SKOV3. Transwell chambers were incubated for 10–12 hours at 37°C. Cell penetration through the membrane was detected by counting the number of cells on the porous membrane after hematoxylin and eosin staining. Images were acquired under the Olympus IX71 inverted microscope (Olympus, Tokyo, Japan).

### 2.7. Xenograft Mouse Model

To assess the effects of IL-6 on EMT *in vivo*, SKOV3 cell xenografts were established in female BALB/c nude mice (aged 6–8 weeks) purchased from the Animal Center of the Academy of Military Medical Sciences (Tianjin, China, production license number: SCXK (Army) 2014-0013). All mice were housed under specific pathogen-free conditions with free access to food and water. Harvested SKOV3 cells (5 × 10^7^) were inoculated subcutaneously into the right axilla, and tumors were established *in situ* approximately three weeks later. Ten nude mice bearing tumors were randomly assigned to an IL-6 group (*n* = 5), and a vehicle control group (*n* = 5). IL-6 was dissolved in 0.1% BSA-PBS, and the animal were dosed by intratumoral injection at 20 *μ*g/50 *μ*L/mouse every 3 days. After six IL-6 injections, mice were sacrificed by cervical dislocation, and the EMT phenotype was analyzed. Visible tumor nodules were excised, weighed, and measured with a caliper to calculate the volume defined as *V* = *π*/6 (*ab*^2^) (*a*, major diameter; *b*, minor diameter). The experiment protocol on BALB/c nude mice was conducted with approval by the Ethics Committee on Animal Experimentation of Tianjin University of Traditional Chinese Medicine (Tianjin, China, protocol number: SCXK 2016-0006), and all procedures involving the animals were conducted according to guidelines and regulations.

### 2.8. Gene Expression Profiles of Publicly Available Databases

The large-scale gene expression profile was retrieved from The Cancer Genome Atlas (TCGA) using cBioPortal (http://www.cbioportal.org/). The publicly available microarray dataset and accompanying clinical information for 489 human tissue specimens of ovarian serous cystadenocarcinoma in TCGA were obtained for analysis [29]. A signature incorporating several relevant markers is superior to any single marker for assessing the complex biologic processes in EMT [30, 31]; therefore, we applied a previously developed EMT scoring system for tumors based on transcriptomic data from TCGA datasets for universal quantification of the EMT state. Generic EMT scores were calculated according to a previously described method [32]. We identified an epithelial gene set consisting of known epithelial markers (E-cadherin, P-cadherin, DDR1, and KRT8) and a mesenchymal gene set consisting of known mesenchymal markers (N-cadherin, vimentin, Snail, Slug, Twist1, ZEB1, ZEB2, and fibronectin-1). Subsequently, principal component analysis of these EMT markers was performed, and the first principal component of each EOC sample was calculated as the EMT score. High EMT scores indicated a mesenchymal phenotype, and low EMT scores indicated a mesenchymal phenotype.

### 2.9. Statistical Analysis

Data shown in the figures are representative of at least three independent experiments *in vitro*. For parametric data, comparison of different groups was performed by one-way analysis of variance (ANOVA), followed by a least squares difference (LSD) test for multiple comparisons. The relationship between IL-6/STAT3/HIF-1*α* mRNA expression and EMT score was explored in 489 EOC patients from TCGA datasets using Spearman's correlation analysis. The data were analyzed by the SPSS version 25.0 for Windows (SPSS Inc., Chicago, IL, USA). *P* < 0.05 was considered to indicate statistical significance.

## 3. Results

### 3.1. IL-6 Stimulates the EMT Phenotype of EOC

**Figure 1:**
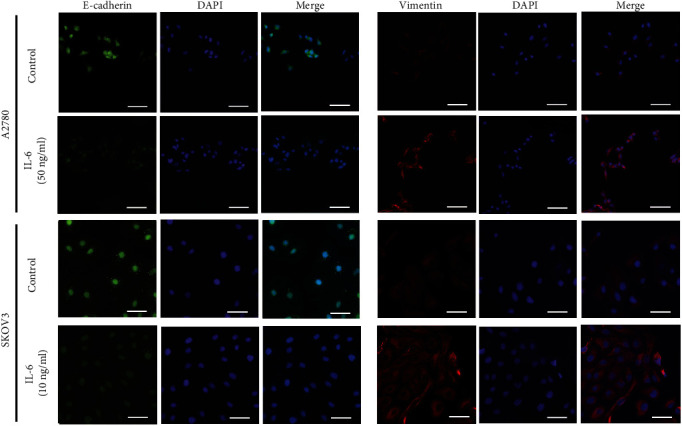
IL-6 promotes the EMT phenotype in EOC cell lines. A2780 cells were treated with exogenous IL-6 (50 ng/ml) for 24 hours, and SKOV3 cells were treated with exogenous IL-6 (10 ng/ml) for 48 hours under normoxic conditions. The expression and localization of E-cadherin and vimentin were then detected by immunofluorescence. The nuclei were stained with DAPI (blue) as an internal reference. E-cadherin and vimentin proteins were visualized by Alexa 488 fluorescent labeling (green) and 549 fluorescent labeling (red), respectively. Scale bars: 100 *μ*m.

Cell lines representing either the epithelial state or the mesenchymal state were required to elucidate the regulatory role of IL-6 on EMT. We evaluated four putative EOC cell lines for the expression of EMT markers at both the mRNA and protein levels (Supplemental Figure S1). A2780 cells derived from human ovarian epidermal tissue have a greater propensity to exhibit the epithelial phenotype, whereas SKOV3 cells derived from cancer cells suspended in ascites tend toward the mesenchymal phenotype. Thus, A2780 and SKOV3 were confirmed as suitable cell models for experimental studies. Owing to our previous study that showed IL-6 secreted by A2780 and SKOV3 cells may enhance their ability of invasion [12], we concentrated on the induction of EMT by IL-6. Immunofluorescence microscopy was utilized to compare immunostaining of E-cadherin and vimentin in EOC cells with and without IL-6 treatment. E-cadherin and vimentin were detected mainly in the cytoplasm, and this localization remained unchanged after IL-6 treatment. Exogenous IL-6 treatment resulted in decreased E-cadherin expression, whereas vimentin was increased in both A2780 and SKOV3 cells (Figure 1). These observations were consistent with those obtained by RT-qPCR and western blot analyses subsequently.

Thereafter, we constructed tumor xenograft mouse models to validate the effect of IL-6 on EMT. The *in vivo* effects of IL-6 on the expression of EMT markers were consistent with those observed *in vitro*. Compared with vehicle control group, the expression of E-cadherin expressed was decreased in the IL-6 injection group, whereas the expression of vimentin, Twist1, and Snail was increased (Figure S2(c)). Combined with these results *in vitro* and *in vivo*, we believe that IL-6 promotes EMT in EOC cells.

### 3.2. Hypoxia Induces EMT to Promote Invasion Directly by HIF-1*α*

Because EMT and invasion are two interdependent biological functions in the metastatic cascade leading to tumor development, we measured the levels of EMT marker proteins and invasion ability simultaneously in the treated EOC cells and their transfectants under hypoxia.

**Figure 2:**
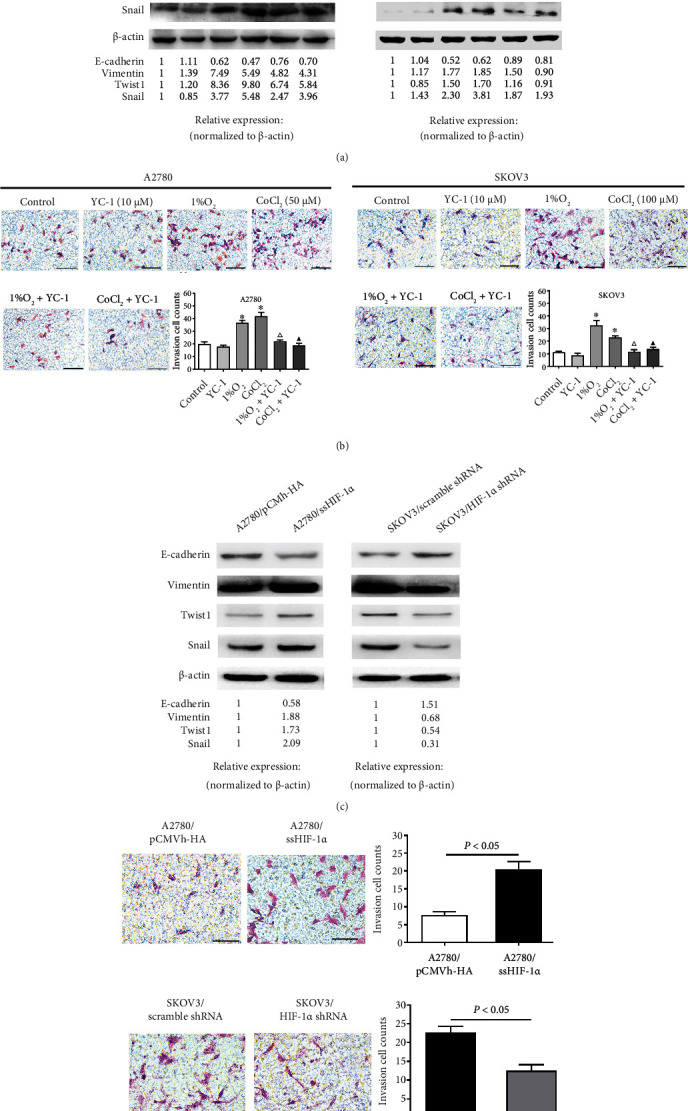
Hypoxia-induced HIF-1*α* regulates the levels of EMT markers and invasion in EOC cell lines. (a) Relative protein levels of EMT markers in A2780 and SKOV3 after YC-1 treatment under normoxic (21% O_2_) or hypoxic (1% O_2_ or CoCl_2_) conditions, respectively. (b) The invasion ability of A2780 and SKOV3 treated as described for (a) was detected by Matrigel invasion assay. Scale bars: 100 *μ*m. In each experiment, the numbers of cells that penetrated the membrane were counted in five microscopic fields per filter. The invasion cell counts are presented as the mean ± SD of three independent experiments. ∗*P* < 0.05 compared with the control group, △*P* < 0.05 compared with the 1% O_2_ group, and ▲*P* < 0.05 comparedwith the CoCl_2_ group by one-way ANOVA with LSD multiple comparison test. (c) Protein levels of EMT markers in transfected A2780 cells overexpressing HIF-1*α* and transfected SKOV3 cells with repression of HIF-1*α* under normoxic conditions. (d) The invasion ability of transfected A2780 cells overexpressing HIF-1*α* and transfected SKOV3 cells with repression of HIF-1*α* was detected by Matrigel invasion assay. Scale bars: 100 *μ*m.

Compared with the control group, 1% O_2_ alone or CoCl_2_ alone treatment promoted the EMT phenotype and cell invasion in both the A2780 and SKOV3 cell lines (Figures 2(a) and 2(b)). Meanwhile, HIF-1*α* inhibitor YC-1 was sufficient to attenuate the development of the EMT phenotype and cell invasion after hypoxia treatment (1% O_2_ and CoCl_2_; Figures 2(a) and 2(b)). Similarly, enhanced expression of HIF-1*α* in A2780 cells promoted the EMT phenotype and invasion ability, whereas repressed expression of HIF-1*α* in SKOV3 cells had the opposite effects (Figures 2(c) and 2(d)). These results suggest that HIF-1*α* located downstream of hypoxia is required for the acquisition of EMT phenotype and invasion ability.

### 3.3. IL-6 and Hypoxia Possess Synergistic Effects on EMT in EOC Cells

**Figure 3:**
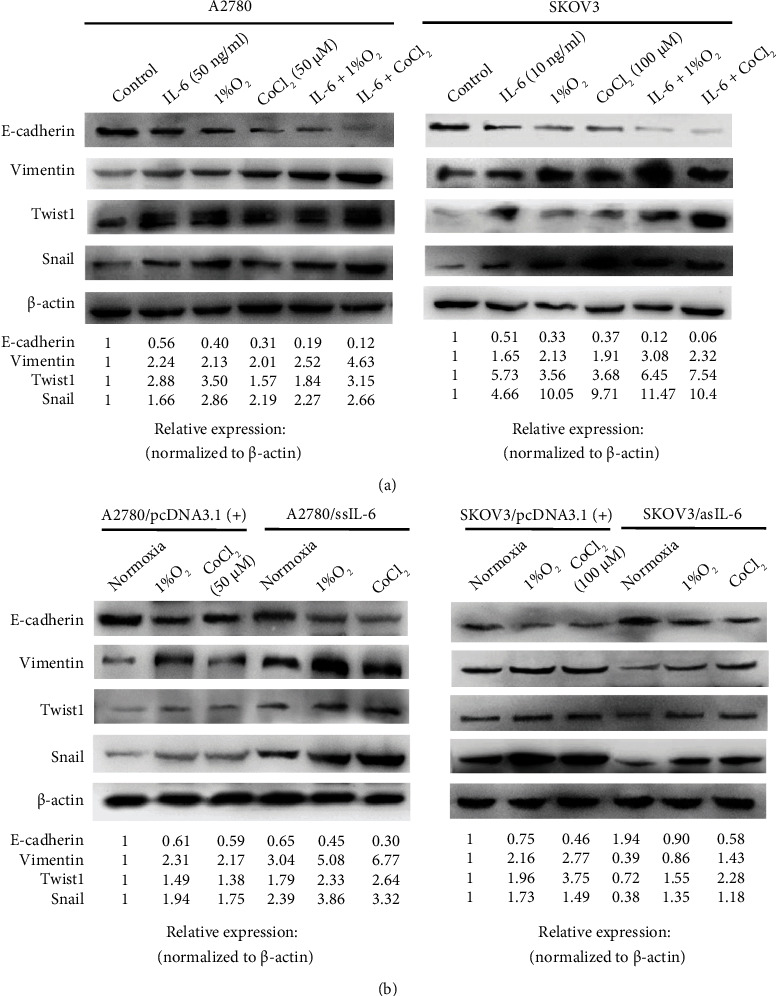
Exogenous and endogenous IL-6 combined with hypoxia regulates the protein expression of EMT markers in EOC cell lines. (a) A2780 and SKOV3 cells were treated with or without IL-6 as described in Figure 1 under normoxic (21% O_2_) or hypoxic (1% O_2_ or CoCl_2_) conditions, respectively. After treatment, the protein levels of EMT markers were analyzed by western blotting. (b) A2780 clones overexpressing IL-6 and SKOV3 clones with depletion of IL-6 were treated under normoxic (21% O_2_) or hypoxic (1% O_2_ or CoCl_2_) conditions as described for (a), respectively. After treatment, the protein levels of EMT markers were analyzed by western blotting. Representative images from three independent experiments are shown.

Treatment with exogenous IL-6 alone or hypoxia (1% O_2_ and CoCl_2_) alone significantly decreased the mRNA level of E-cadherin, but increased the levels of vimentin, Twist1, and Snail compared with those in the control group in both A2780 and SKOV3 cells (Figure S3(a)). As illustrated in Figure 3(a), the change trends in EMT markers at the protein level were consistent with those at the mRNA level. The combination of exogenous IL-6 and 1% O_2_ or CoCl_2_ clearly diminished E-cadherin expression and enhanced vimentin expression at both the mRNA and protein levels, and the extent of this modulation was higher than induced by either treatment alone in both cell lines. Although the combination of exogenous IL-6 and hypoxia also increased the expression of the three EMT-TFs, the synergistic effect was less apparent in most cases. It can be speculated that exogenous IL-6 and hypoxia function synergistically to preferentially alter the activity rather than expression of these EMT-TFs. Considering that E-cadherin and vimentin reflect the EMT phenotype more directly than EMT-TFs, we generally accepted the synergistic effects of IL-6 and hypoxia on stimulating EMT.

To provide established cell models, IL-6 was stably overexpressed in the A2780 cell line and repressed in the SKOV3 cell line as previously described [28]. As illustrated in Figure 3(b) and Supplemental Figure S3(b), E-cadherin was downregulated at both the mRNA and protein levels, whereas vimentin, Twist1, and Snail were upregulated in ssIL6-transfected A2780 cells compared with the corresponding control-vector-transfected cells under normoxic, hypoxic and simulated hypoxic conditions. In stark contrast, stable suppression of IL-6 in SKOV3 cells resulted in an opposite pattern of alterations in the expression of EMT markers. Interestingly, the degree of changes in the expression of EMT markers indicated that the transfected cell lines exhibited higher sensitivity to the synergistic effects of IL-6 and hypoxia.

### 3.4. HIF-1*α* Is a Vital Mediator of IL-6-Induced EMT and Invasion

**Figure 4:**
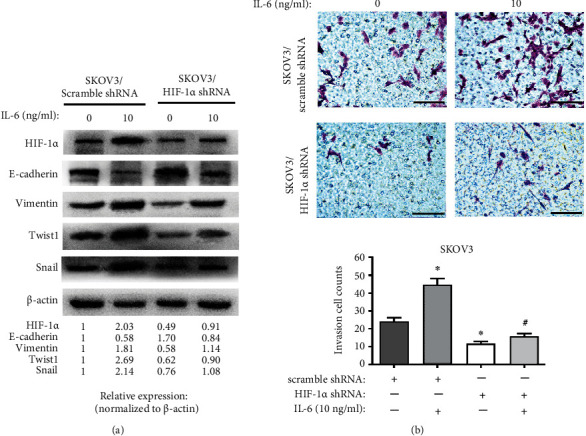
HIF-1*α* inhibits the induction effect of IL-6 on EMT and invasion. (a) Protein levels of EMT markers in transfected SKOV3 cells with repression of HIF-1*α* after treatment with exogenous IL-6 (10 ng/ml) for 48 hours under normoxic conditions. (b) The invasion ability of transfected SKOV3 cells with repression of HIF-1*α* was detected by Matrigel invasion assay after treatment as described for (a). Scale bars: 100 *μ*m. In each experiment, the numbers of cells that penetrated the membrane were counted in five microscopic fields per filter. The invasion cell counts are presented as the mean ± SD of three independent experiments. ∗*P* < 0.05 compared with the SKOV3/scramble shRNA group and #*P* < 0.05 compared with the IL-6-treated SKOV3/scramble shRNA group by one-way ANOVA with LSD multiple comparison test.

To define the role of HIF-1*α* in IL-6-induced EMT, we determined the combined effects of IL-6 treatment and shRNA-mediated interference of HIF-1*α* on EOC cells. Since the change ranges of EMT markers and invasion ability caused by exogenous IL-6 in SKOV3 cells were bigger than those in A2780 cells (Figure 3), as well as the SKOV3 cells in normal state containing higher levels of HIF-1*α* when compared with the A2780 cells [33], we chose SKOV3 cells for the HIF-1*α* silence experiment. HIF-1*α* downregulation significantly inhibited the IL-6-induced EMT phenotype and cell invasion in SKOV3 as compared with the IL-6-treated SKOV3/scramble shRNA group (Figure 4). These results implicated HIF-1*α* as a critical factor in the process of EOC progression driven by IL-6.

### 3.5. IL-6 Induces EMT of EOC Cells via IL-6/STAT3/HIF-1*α* Loop

**Figure 5:**
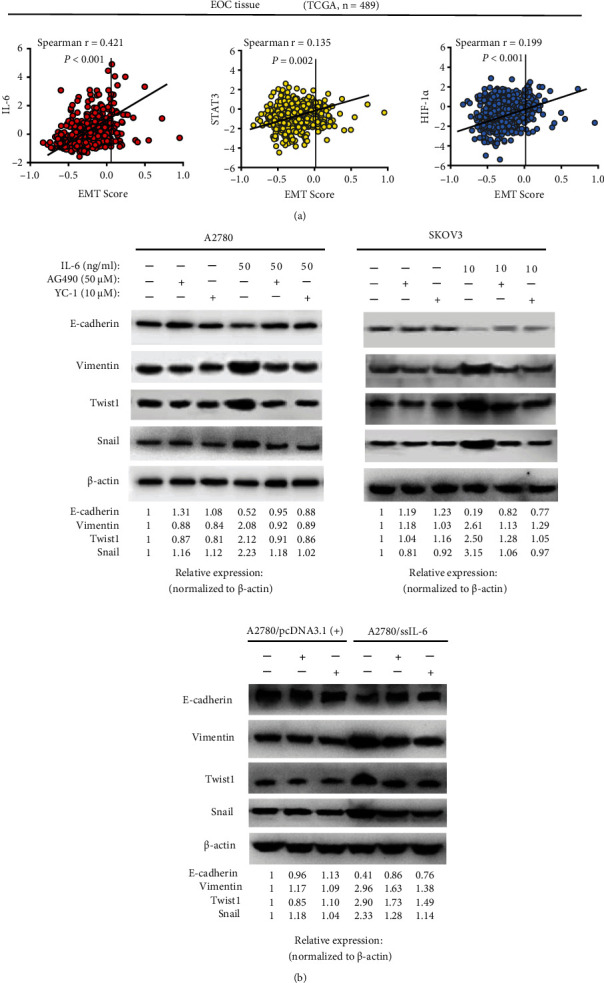
The IL-6/STAT3/HIF-1*α* loop mediates IL-6-induction EMT in EOC. (a) Correlation between the generic EMT score and gene expression of each molecule in the signaling loop. Scatter plots showed EMT signature scores and normalized mRNA levels of 489 EOC samples based on TCGA dataset. Spearman's rank correlation analysis. (b) Blocking STAT3 and HIF-1*α* with corresponding inhibitors (AG490 inhibits STAT3 and YC-1 inhibits HIF-1*α*) reversed IL-6-induced EMT in A2780, SKOV3, and A2780 clones overexpressing IL-6. Protein levels displayed are representative of three independent experiments with similar results.

To gain an insight into the molecular mechanism of IL-6-induced EMT and the interaction with HIF-1*α*, we first utilized generic microarrays to quantify the correlation of EMT signatures and underlying molecules involved in the mediation of EMT status. In 489 EOC patients retrieved from TCGA datasets, positive correlations were observed between EMT score and the mRNA expression of each signaling molecule in IL-6/STAT3/HIF-1*α* loop (Figure 5(a)).

To future ascertain the influence of the IL-6/STAT3/HIF-1*α* loop in EMT, we blocked components of the loop, respectively, by pretreatment with specific inhibitors. Since EMT markers function at the posttranslational level and it is not clear whether the loop affects EMT marker expression in EOC cells through directed transcriptional regulation, we detected the expression of EMT marker proteins in blocking studies. As illustrated in Figure 5(b), the inhibitors of STAT3 (AG490) and HIF-1*α* (YC-1) reversed the process of exogenous IL-6 induced EMT to varying degrees but had no significant effects on negative control cells. These inhibitors also impaired the response to endogenous IL-6, confirming that IL-6 may induce EMT in EOC cells via the IL-6/STAT3/HIF-1*α* loop.

## 4. Discussion

Recent studies have provided growing insights into IL-6 as a crossroads in immune responses and tumorigenic potential. Previous studies conducted by our group [12] and others [34] have shown that IL-6 promotes EOC cell proliferation, adhesion, and invasion. Those findings need to be complemented and extended. In view of hypoxia as an important modulator of inflammation and immune responses, we assessed the cross-influence of IL-6 and hypoxia on EMT markers in two EOC cell lines (A2780 and SKOV3) and their stably transfected cell clones. Remarkably, we discovered that IL-6 and hypoxia synergistically promote EMT by activating the IL-6/STAT3/HIF-1*α* loop, which may reveal a part of the complex regulatory network mediated by inflammatory and hypoxic microenvironment in EOC progression.

EMT program engaged at sites of inflammation in an experimental model of cancer [35]. Chronic inflammatory mediators induce the expression of transcription factors that repress the epithelial phenotype and promote tumor EMT. Epithelial cells in numerous cancer types have been shown to undergo EMT and acquire an invasive morphological phenotype following exposure to IL-6 [14–16]; however, the potential pathways of induction of EMT in EOC are not well understood. It appears that the effect of IL-6 on EMT may depend on the tumor cell type. Colomiere et al. [36] indicated that EGFR-induced crosstalk with the IL-6R pathway may result in the acquisition of some EMT-associated phenotypes in ovarian cancer, although emphasis was placed on EGFR-mediated STAT3 activation, and the evidence for IL-6-induced EMT was incomplete.

Hypoxia is a factor of the microenvironment in almost all tumor types and is known to upregulate multiple mediators and pathways that induce tumor EMT [37]. The anatomical position deep in the pelvic cavity and rapid proliferation in malignancy make the hypoxic microenvironment especially apparent in EOC. As an innovation, in this study, we investigated the reciprocal action of hypoxia and IL-6 on inducing EMT. The results showed that both 1% O_2_ and CoCl_2_ treatments not only promote EMT alone, but also assisted IL-6 to enhance the inductive function of EMT. HIF-1*α*, a key element in regulating oxygen homeostasis, is mainly associated with hypoxia-related diseases (such as ischemia, pulmonary hypertension, and cancer) and the pathogenesis of inflammation. Our previous study verified the positive correlation existed between HIF-1*α* level and EOC malignant progression in clinical specimens [33]. Intratumoral hypoxia conditions can activate and stabilize the HIF-1*α* transcription complex, which regulates Twist or Snail, two key EMT regulators, and, thereby, enhances tumor aggressiveness [38, 39]. The functions of HIF-1*α* in EOC cells were analyzed emphatically by transferable plasmids encoding or silencing HIF-1*α* as well as the pharmacological inhibitor of HIF-1*α* (YC-1). Our subsequent experiments demonstrated that HIF-1*α* is an important cofactor that mediates IL-6-induced EMT and cell invasion.

**Figure 6:**
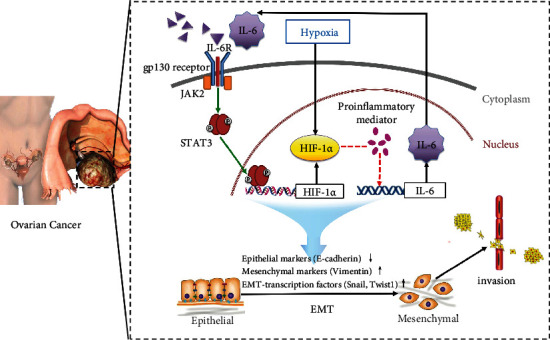
Schematic of the IL-6/STAT3/HIF-1*α* autocrine signaling loop that mediates the interaction between IL-6 and HIF-1*α* in the acquisition of EMT and invasion under hypoxic conditions. Solid lines indicate a direct action and dotted lines show an indirect action. IL-6 in the tumor microenvironment binds to its receptor and thereupon triggers the phosphorylation and nuclear localization of STAT3 by Janus kinase 2. Activated STAT3 dimers bound to specific sites in target gene promoters inducing transcription of HIF-1*α*. In turn, HIF-1*α* facilitates IL-6 production indirectly through proinflammatory mediators, such as NF-*κ*B, COX-2, or TLR4. More IL-6 is secreted out of EOC cells, further reinforcing this loop. Among these components of the loop, STAT3 and HIF-1*α* have been shown to regulate the expression of EMT markers as well as EMT-related transcription factors, leading to the invasion and metastasis of EOC cells.

It is significant to clarify the mechanism responsible for the synergistic effects of IL-6 and hypoxia on EMT. Signal transducer and activator of transcription proteins (STATs) has been identified as an essential mediator of the inflammation and hypoxia [40]. STATs will be activated after binding to their receptors of certain growth factors or cytokines, such as IL-6 [41]. In particular, STAT3 has been reported to stabilize HIF-1*α* and avoid its degradation. Even independently of hypoxia, STAT3 induces HIF-1*α* gene transcription and, in consequence, accelerates its synthesis [42]. Based on published data demonstrating that the IL-6/STAT3 axis accelerates EMT and leads to further tumor progression [17, 18, 36]. Meanwhile, Cho et al. [43] found that STAT3 could upregulate Twist1 expression by directly binding to the Twist1 promoter and promote prostate cancer cell invasion through the STAT3/HIF-1*α*/Twist1 signaling cascade. Therefore, we focused on the participation of STAT3 signaling pathways as the main components that interconnect IL-6 and hypoxia. A novel signaling cascade, IL-6/STAT3/HIF-1*α*, has been discovered in numerous cancer types including pancreatic cancer, prostate cancer, lung cancer, colon cancer, and malignant glioma [44, 45]. Given that we have confirmed IL-6/STAT3/HIF-1*α* can form a positive feedback loop in ovarian cancer [27], we propose that this loop is involved in the interconnection between two induction factors of EMT, IL-6, and hypoxia, as depicted in Figure 6.

Our data mining results of 489 EOC patients from TCGA datasets foreshadowed the inspiring associations between EMT and the inflammatory signaling loop. It should be noted that blockade of neither STAT3 (AG490) nor HIF-1*α*(YC-1) completely inhibited EMT in EOC cells. We speculated that other mechanisms mediated by IL-6 exert influence on the induction of EMT. In fact, noncoding RNAs, in particular post-transcriptional microRNA regulatory loops linked to IL-6, may contribute to EMT plasticity and support cancer cell invasion [41, 46, 47]. Another approach to the co-stimulation of IL-6 and HIF-1*α* in EOC cells could be toll-like receptor 4 (TLR4)/NF-*κ*B/HIF-1*α* regulatory feedback circuit [33]. As a core downstream component of the TLR4 signaling pathway, NF-*κ*B has been shown to directly bind to the HIF-1*α* promoter via its subunits p50 and p65 [48, 49]. Not only that, TLR4-mediated NF-*κ*B after activation of lipopolysaccharide, or paclitaxel inflammatory promoted cytokines including IL-8 and IL-6 [50]. These show that the biological processes in EOC involved in the cross-communication between inflammation and hypoxia are complex, and the link with IL-6 and hypoxia requires further investigation.

Regrettably, due to the technical limitations of transplanted tumor in nude mouse, this study established only a small number of xenograft models fully and lacked the phenotype of metastatic spread *in vivo*, despite of the superficial measurement of EMT markers with exogenous IL-6 treatment in EOC mouse models. EMT has been recognized as a pro-metastatic cellular event, and the conclusions could be more powerful if any evidence of metastatic behavior is demonstrated. In addition, the chemical inhibitors targeting STAT3 and HIF-1*α* were dissolved in dimethyl sulfoxide, which is toxic and not suitable for use in the tumor xenograft model. Therefore, blockade experiments of the inflammatory signaling loop were not performed *in vivo*.

## 5. Conclusions

Our study explored the synergistic effect and its molecular mechanism that IL-6 and hypoxia induce EMT to promote invasion in two EOC cell lines. The current results provided strong evidence supporting an interaction exists between IL-6 and HIF-1*α* induced by hypoxia in the acquisition of EMT and invasion. Furthermore, IL-6-induced EMT by either paracrine or autocrine was reversed through STAT3 and HIF-1*α* blockade, which implicated the IL-6/STAT3/HIF-1*α* loop we have confirmed the presence of EOC may be involved in cross-communication infiltration and hypoxia, and facilitate EOC progression and invasiveness. These findings provide new directions for EOC therapy by targeting inflammation signaling pathways and would enhance the efficacy of immunotherapy.

## Data Availability

Data supporting this research article are available from the corresponding author or first author on reasonable request.

## Supplementary Materials

Supplementary Materials Figure S1: Comparison of initial EMT status in EOC cell lines under normoxic conditions. Relative mRNA (above) and protein (below) expressions of E-cadherin, vimentin, Twist1, and Snail in in four EOC cell lines, A2780, SKOV3, OVCAR3v and ES-2 cells were measured by RT-qPCR and western blot analysis, respectively. Figure S2: IL-6 regulates the levels of EMT markers expressed in xenografts of EOC mouse models. The EOC mouse model was constructed by injection of SKOV3 cells into the right axilla of nude mice. After six injections of IL-6, mice were sacrificed and analyzed as follows: (a) representative photos and HE-stained images of tumor nodules. Scale bars: 250 *μ*m. (b) Tumor volume and tumor weight in the IL-6 group (*n* = 5) and control group (*n* = 5) were compared at the end of the study by Mann–Whitney *U*-test. (c) Relative mRNA and protein expressions of EMT markers in tumor nodules were analyzed by RT-qPCR and western blotting, respectively. Figure S3: IL-6 regulates mRNA expression of EMT markers *in vitro*. (a) A2780 cells were treated with exogenous IL-6 (50 ng/ml) for 24 hours and SKOV3 cells were treated with exogenous IL-6 (10 ng/ml) for 48 hours under normoxic (21% O_2_) or hypoxic (1% O_2_ or CoCl_2_) conditions, respectively. (b) A2780 clones overexpressing IL-6 and SKOV3 clones with depletion of IL-6 were treated under normoxic (21% O_2_) or hypoxic (1% O_2_ or CoCl_2_) conditions as described for (a), respectively. After treatment, the mRNA levels of EMT markers were analyzed by RT-qPCR.
