# Supplementary material for: Interleukin-6 and Hypoxia Synergistically Promote EMT-Mediated Invasion in Epithelial Ovarian Cancer via the IL-6/STAT3/HIF-1α Feedback Loop
Source: Anal Cell Pathol (Amst). 2023 Feb 13;2023:8334881. doi: 10.1155/2023/8334881 (PMC9940980; doi:10.1155/2023/8334881)
Supplement: Supplementary Materials — Figure S1: Comparison of initial EMT status in EOC cell lines under normoxic conditions. Relative mRNA (above) and protein (below) expressions of E-cadherin, vimentin, Twist1, and Snail in in four EOC cell lines, A2780, SKOV3, OVCAR3v and ES-2 cells were measured by RT-qPCR and western blot analysis, respectively. Figure S2: IL-6 regulates the levels of EMT markers expressed in xenografts of EOC mouse models. The EOC mouse model was constructed by injection of SKOV3 cells into the right axilla of nude mice. After six injections of IL-6, mice were sacrificed and analyzed as follows: (a) representative photos and HE-stained images of tumor nodules. Scale bars: 250 μm. (b) Tumor volume and tumor weight in the IL-6 group (n = 5) and control group (n = 5) were compared at the end of the study by Mann–Whitney U-test. (c) Relative mRNA and protein expressions of EMT markers in tumor nodules were analyzed by RT-qPCR and western blotting, respectively. Figure S3: IL-6 regulates mRNA expression of EMT markers in vitro. (a) A2780 cells were treated with exogenous IL-6 (50 ng/ml) for 24 hours and SKOV3 cells were treated with exogenous IL-6 (10 ng/ml) for 48 hours under normoxic (21% O2) or hypoxic (1% O2 or CoCl2) conditions, respectively. (b) A2780 clones overexpressing IL-6 and SKOV3 clones with depletion of IL-6 were treated under normoxic (21% O2) or hypoxic (1% O2 or CoCl2) conditions as described for (a), respectively. After treatment, the mRNA levels of EMT markers were analyzed by RT-qPCR. [file 8334881.f1.docx]

**Supplementary figures**


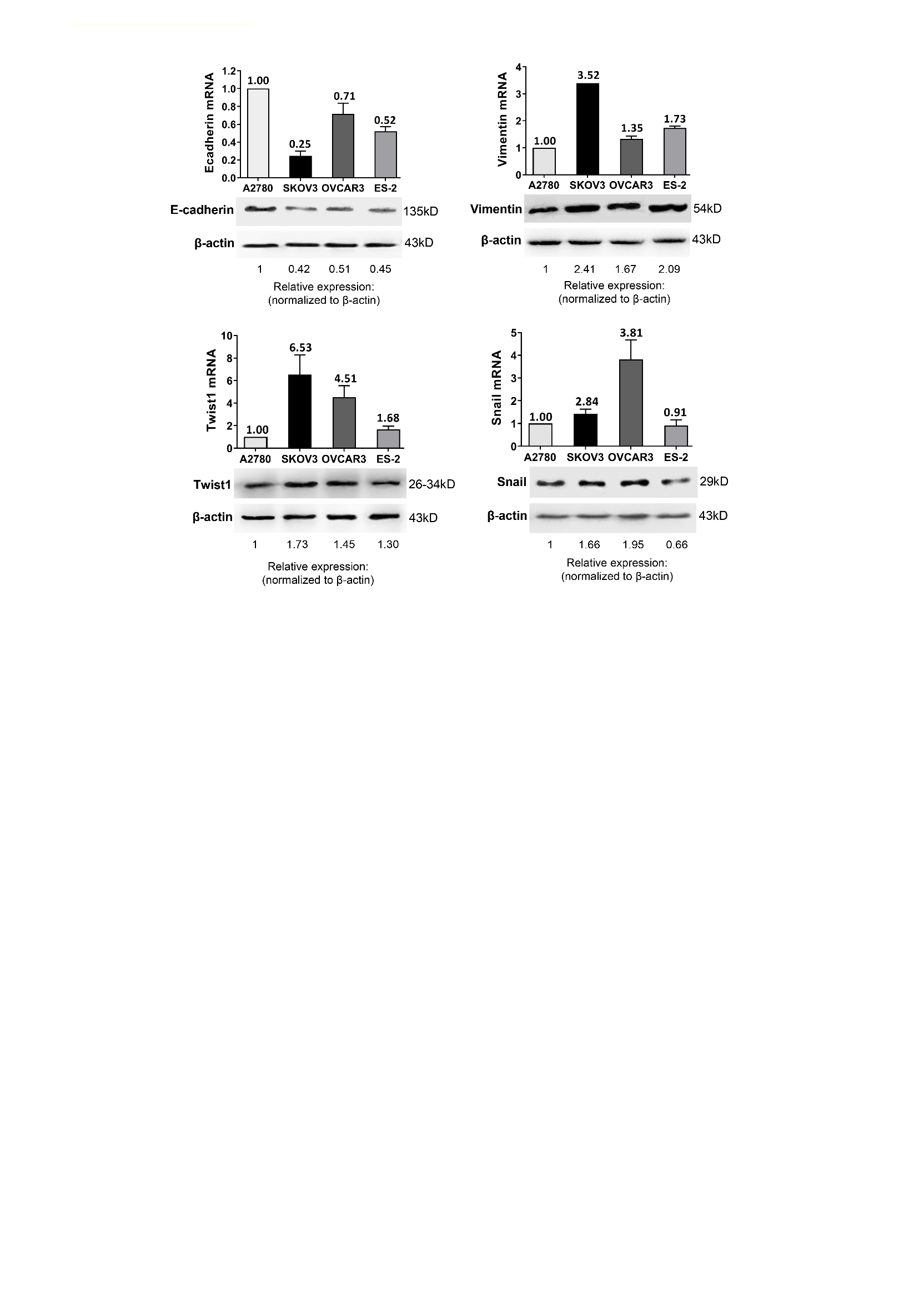


**Figure S1.** Comparison of initial EMT status in EOC cell lines under normoxic conditions.

Relative mRNA (above) and protein (below) expressions of E-cadherin, Vimentin, Twist1 and Snail in in four EOC cell lines, A2780, SKOV3, OVCAR3 and ES-2 cells were measured by RT-qPCR and Western blot analysis, respectively. Three independent experiments were performed. Data were presented as mean ± standard deviation (SD). Representative images from three independent experiments are shown.

【Expand analysis】As shown in Figure S1, expression levels of EMT markers at the protein level were broadly similar to those at the mRNA level. E-cadherin is expressed at high levels in A2780 cells, while the expression of other EMT markers, especially Snail and Twist1, was dramatically decreased in A2780 cells. The pattern of expression of these markers was similar in ES-2 cells. Among the four human ovarian cancer cell lines investigated, SKOV3 cells expressed the lowest levels of E-cadherin but the highest levels of Vimentin. In contrast, OVCAR-3 cells expressed low levels of E-cadherin and Vimentin and high levels of Snail and Twist1.

Furthermore, our previous studies confirmed SKOV3 cells secrete far more IL-6 than any other human ovarian cancer cell lines^[1]^. Thus, A2780 and SKOV3 were used in our subsequent functional studies. Thus, A2780 and SKOV3 were used in our subsequent functional studies.

[1] Y Wang, XL Niu, Y Qu, J Wu, YQ Zhu, WJ Sun, LZ Li, Autocrine production of interleukin-6 confers cisplatin and paclitaxel resistance in ovarian cancer cells, Cancer Lett 295 (2010) 110-123, <https://doi.org/10.1016/j.canlet.2010.02.019>.


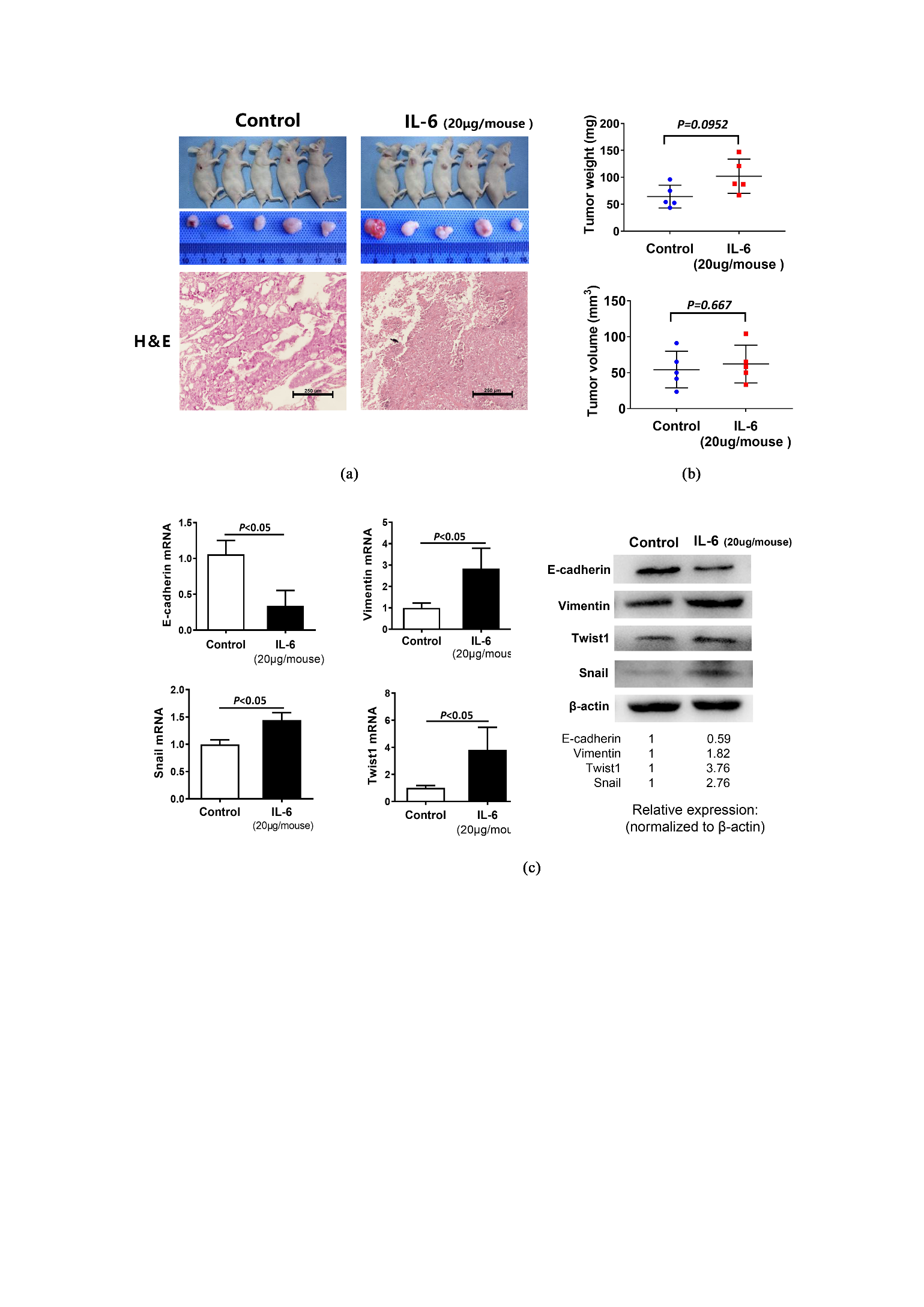


**Figure S2.** IL-6 regulates the levels of EMT markers expressed in xenografts in EOC mouse models.

The EOC mouse model was constructed by injection of SKOV3 cells into the right axilla of nude mice. After six injections of IL-6, mice were sacrificed and analyzed as follows: (a) Representative photos and HE-stained images of tumor nodules. Scale bars: 250 μm. (b) Tumor volumes and tumor weights in the IL-6 group (n = 5) and control group (n = 5) were compared at the end of the study by Mann–Whitney U-test. (c) Relative mRNA and protein expressions of EMT markers in tumor nodules were analyzed by RT-qPCR and Western blotting, respectively. Data represent the mean ± SD.

【Expand analysis】Because of the low rate of tumor formation following transplantation of A2780 cells, athymic nude mice were inoculated only with SKOV3 cells. The typical histological structure of a cystadenocarcinoma was confirmed pathologically by microscopic examination of H&E-stained tissues, indicating that the tumor xenograft model was successfully established (Figure S2(a)). The parenchymal cells were more densely arranged in the tumors in the IL-6 group, probably due to the effects of IL-6 as a proliferative factor.


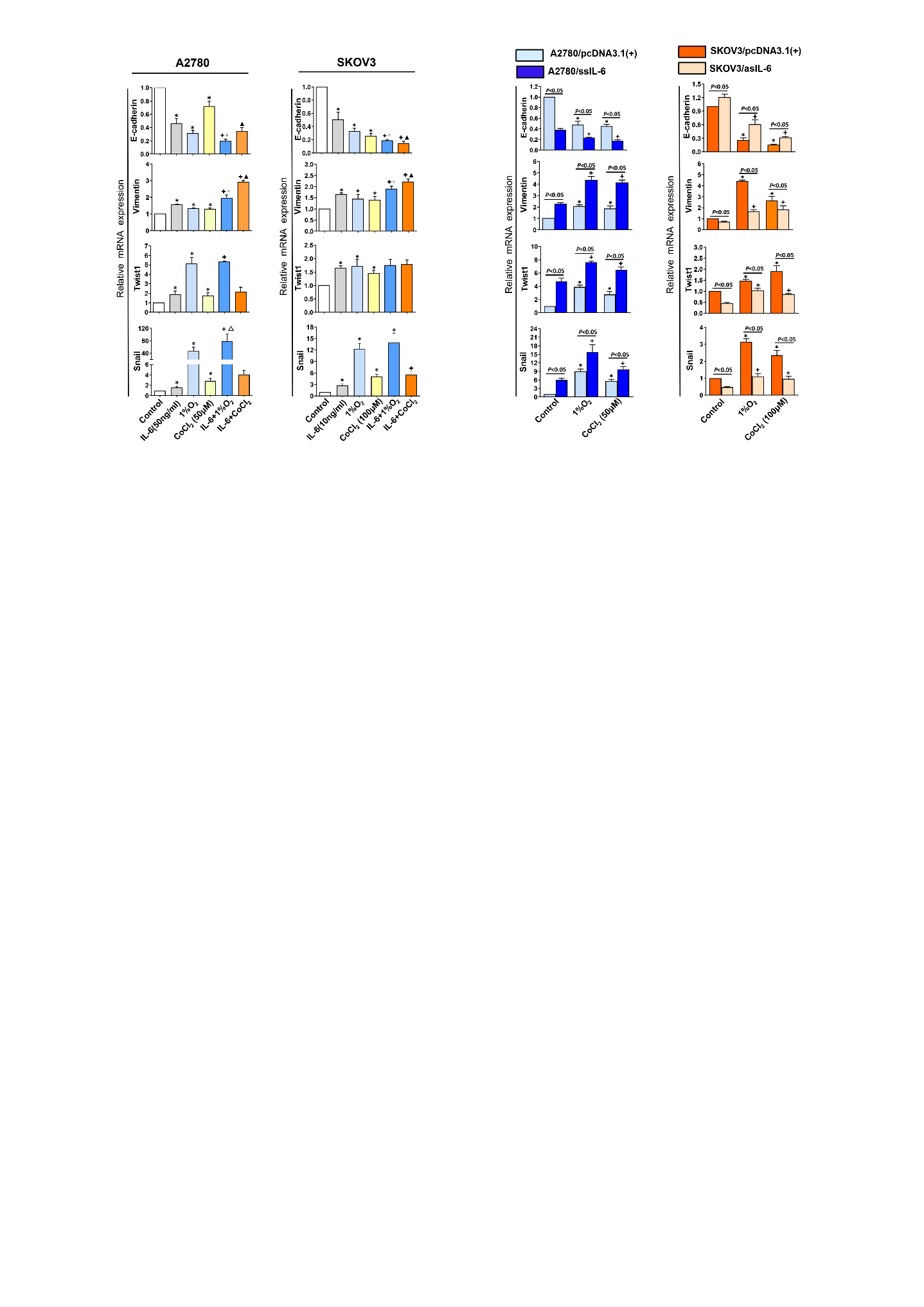


**Figure S3.** IL-6 regulates mRNA expressions of EMT markers *in vitro*.

(a) A2780 cells were treated with exogenous IL-6 (50 ng/ml) for 24 h and SKOV3 cells were treated with exogenous IL-6 (10 ng/ml) for 48 h under normoxic (21% O_2_) or hypoxic (1% O_2_ or CoCl_2_) conditions, respectively. (b) A2780 clones overexpressing IL-6 and SKOV3 clones with depletion of IL-6 were treated under normoxic (21% O_2_) or hypoxic (1% O_2_ or CoCl_2_) conditions as described for (a), respectively. After treatment, mRNA levels of EMT markers were analyzed by RT-qPCR. Data represent the mean ± SD of three independent experiments. For (a): **P* < 0.05 compared to the control group, +*P* < 0.05 compared to the IL-6 group, △*P* < 0.05 compared to the 1%O_2_ group and ▲*P* < 0.05 compared to the CoCl_2_ group by one-way ANOVA with LSD multiple comparisons test. For (b): **P* < 0.05 compared to the A2780/pcDNA3.1(+) in the normoxic cluster and +*P* < 0.05 compared to the SKOV3/pcDNA3.1(+) in the normoxic cluster by one-way ANOVA with LSD multiple comparisons test.
